# Identification of the superior genotypes of pomegranate (*Punica* *granatum* L.) using morphological and fruit characters

**DOI:** 10.1002/fsn3.2450

**Published:** 2021-07-03

**Authors:** Ali Khadivi, Marzieh Arab

**Affiliations:** ^1^ Department of Horticultural Sciences Faculty of Agriculture and Natural Resources Arak University Arak Iran

**Keywords:** aril color, breeding, genetic resource, pomegranate, seed hardness

## Abstract

Pomegranate (*Punica granatum* L.) fruits can be used for fresh consumption, industrial processing, and medicinal purposes. Therefore, it is necessary to evaluate the diversity of its different genotypes to be aware of their potential. In the present study, morphological and pomological diversity of 70 native pomegranate genotypes was evaluated to introduce superior selections. Most of the characters showed significant differences among the genotypes. Fruit weight ranged from 103.28 to 407.59 g, and total aril weight per fruit ranged from 51.55 to 238.97 g. Fruit peel color was highly variable and included yellow, yellow‐red, red, and red‐brown. The sunburn and cracking disorders were not observed on the peel of the majority of genotypes. Aril color was highly variable, including light milky, pink, white‐red, red, and red‐black. Seed was soft in 17, semi‐soft in 21, and hard in 32 genotypes. Total aril weight per fruit was positively and significantly correlated with fruit length (*r* = 0.64), fruit diameter (*r* = 0.87), fruit weight (*r* = 0.95), fruit stalk diameter (*r* = 0.52), fruit peel weight (*r* = 0.71), and aril shape (*r* = 0.32). Principal component analysis (PCA) showed that the fruit‐related traits were important for determining differences between genotypes. Based on the ideal values of commercial characters of pomegranate, 15 genotypes were promising and thus could be directly cultivated in the orchards and used in the breeding programs.

## Graphical abstract

Based on the ideal values of commercial characters of pomegranate, 15 pomegranate genotypes were promising and thus could be directly cultivated in the orchards and used in the breeding programs.

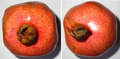

## 1 INTRODUCTION

Pomegranate (*Punica granatum* L., Punicaceae family) is one of the ancient domesticated fruits. Nikolai Vavilov reported its geographical area in the primary Center IV, which includes Asia Minor, Transcaucasia, Iran, and Turkmenistan (Melgarejo & Salazar, 2003). Due to the medicinal and therapeutic properties of pomegranate, this fruit has found high economic value among farmers, which is why its cultivated area has increased in Iran, India, China, Turkey, and the United States (Melgarejo‐Sanchez et al., 2015). Pomegranate adaptation to different climatic and soil conditions has made its distribution and cultivation easier to expand. However, to produce high quality and quantity fruit, this plant needs high temperature during fruit ripening (August to October) (Karimi & Mirdehghan, 2013).

Pomegranate is a valuable fruit that has been used for human food and health since ancient times. Recently, more attention has been paid to this fruit, which has led to the development of cultivation methods, food applications, and its transfer and storage techniques. Pomegranate fruit is considered by consumers due to its organoleptic properties. Pomegranate fruit quality is a balance between taste‐related properties (such as sugars and organic acids) and nutraceutical compounds (such as polyphenols) (Legua et al., 2016; Viuda‐Martos et al., 2010). These innovations have led to the development, consumption, and commercialization of this fruit over the past decades. The diversity of pomegranate genotypes and landraces in the world is high in fruit‐related characteristics, including variation in fruit size, peel color, juice taste and color, seed hardness, resistance to pests and diseases, and the time required from flowering to ripening. The edible part of pomegranate fruit is arils, which constitute about 50% of the whole fruit. The variability of aril‐related traits among different pomegranate genotypes is high so that different genotypes can have arils with diversity in taste, color, size, and seed hardness (Barone et al., 2001). Due to the increasing demand for pomegranate consumption, it is necessary to identify genotypes with ideal fruit quantity and quality with high commercial value (Martinez et al., 2006). Commercially developed pomegranate cultivars in different countries have been selected from local populations (Holland & Bar‐Yakov, 2008), such as “Wonderful” in the United States, “Hicaznar” in Turkey, and “Mollar de Elche” in Spain (Stover & Mercure, 2007).

Genetic diversity in crops, which is essential for food security, the environment, and sustainable development, is being lost. Due to the attention of many countries and producers, the loss of genetic diversity has become a socio‐economic, ethical, and political issue. Therefore, the loss of genetic diversity in crops due to commercialization requires the protection of the existing gene pool not only for the long survival of the species but also for breeding programs. The evolution of powerful new efficient methods for conservation and use of genetic resources has been considered, which in some cases has been beneficial (Esquinas‐Alcazar, 2005). The first step in describing genetic resources and introducing them for use in the production chain, and protecting them, is morphological assessments that provide valuable information about their phenotypic diversity (Frankel, 1970). In the present study, morphological and pomological diversity of native pomegranate genotypes at a collection was evaluated to introduce superior selections.

## 2 MATERIALS AND METHODS

### 2.1 Plant material

Morphological and pomological diversity of 70 native pomegranate genotypes was evaluated to introduce superior selections at a collection in the Siab area from Koodasht region/Lorestan province/Iran. The Siab area is located at 33˚30'44"N latitude, 47˚41'32"E longitude, and 1,196 m height above sea level. The genotypes were named based on the Siab area and numbered from Siab‐1 to Siab‐70. The genotypes were between 10 and 12 years old and were healthy and in full fruiting stage. The orchard management operations, including nutrition, irrigation, and pest and disease control, were performed regularly and uniformly for the genotypes.

### 2.2 Morphological and pomological evaluations

Fifty morphological and pomological traits were used to evaluate phenotypic diversity and to select superior genotypes (Table 1). A total of 20 adult leaves and 20 mature fruits per genotype were randomly selected and harvested. The traits related to dimensions of leaf, fruit, aril, and seed were measured using a digital caliper. A digital scale with an accuracy of 0.01 g was used to measure the weight of fruit, peel, and aril. The qualitative traits (Table 2) were visually examined and coded using the method introduced by Mars and Marrakchi (1999). The total soluble solids (TSS) were determined using a refractometer (pocket PAL‐1 ATAGO Corporation) and expressed in ˚Brix.

**TABLE 1:** Descriptive statistics for the morphological traits utilized in the studied pomegranate genotypes

No.	Character	Abbreviation	Unit	Min.	Max.	Mean	*SD*	CV (%)
1	Tree growth habit	TGH	Code	1	5	3.74	1.53	40.88
2	Tree growth vigor	TGV	Code	3	5	4.43	0.91	20.54
3	Shoot color	ShC	Code	1	7	2.37	1.69	71.18
4	Tree height	The	Code	1	5	4.03	1.26	31.32
5	Trunk type	TrTy	Code	1	3	2.51	0.86	34.42
6	Trunk diameter	TrDi	Code	1	5	2.83	0.74	26.22
7	Canopy density	CaDe	Code	3	5	4.86	0.52	10.68
8	Tendency to produce suckers	TeSu	Code	1	5	2.06	1.59	77.04
9	Leaf length	LLe	mm	43.64	79.81	60.53	8.78	14.50
10	Leaf width	LWi	mm	11.95	20.79	15.78	2.10	13.30
11	Leaf upper surface color	LUSuC	Code	1	3	2.40	0.92	38.46
12	Leaf lower surface color	LLoSuC	Code	1	3	1.49	0.86	57.99
13	Petiole length	PeLe	mm	3.15	7.61	5.06	1.03	20.40
14	Petiole diameter	PeDi	mm	0.34	1.44	1.06	0.20	18.78
15	Yield	Yi	Code	3	5	4.71	0.71	14.97
16	Fruit length	FrLe	mm	70.37	113.30	81.92	6.60	8.06
17	Fruit diameter	FrDi	mm	56.72	90.20	65.72	5.37	8.17
18	Fruit weight	FrWe	G	103.28	407.59	158.04	42.40	26.83
19	Fruit stalk length	FrStLe	mm	4.47	21.51	10.88	2.82	25.89
20	Fruit stalk diameter	FrStDi	mm	2.07	6.80	3.47	0.70	20.04
21	Fruit shape	FrSh	Code	1	3	1.34	0.76	56.64
22	Fruit symmetry	FrSy	Code	0	1	0.79	0.41	52.28
23	Stamen density in fruit calyx	StDe	Code	1	5	3.46	1.60	46.33
24	Fruit calyx form	FrCalFo	Code	1	5	4.09	1.31	31.91
25	Fruit peel color	FrPeC	Code	1	7	3.34	1.56	46.68
26	Fruit peel sunburn presence	FrPeSu	Code	0	3	0.67	1.16	173.73
27	Fruit peel cracking presence	FrPeCr	Code	0	1	0.04	0.20	510.00
28	Fruit peel thickness	FrPeTh	Code	1	5	2.40	1.38	57.38
29	Sepal number	SepNo	Number	5.40	6.60	5.88	0.25	4.19
30	Sepal length	SepLe	mm	9.56	21.51	12.81	1.93	15.05
31	Sepal base width	SepBWi	mm	0.44	1.14	0.79	0.17	21.25
32	Fruit calyx length	FrCalLe	mm	11.72	30.22	19.29	3.64	18.88
33	Fruit calyx diameter	FrCalDi	mm	10.20	24.75	15.02	3.34	22.24
34	Fruit peel weight	FrPeWe	G	40.14	156.83	68.54	17.11	24.96
35	Internal peel color	InPeC	Code	1	3	1.37	0.78	57.15
36	Septum color	SeptC	Code	1	5	2.89	1.36	46.96
37	Septum thickness	SeptTh	Code	1	5	2.49	1.21	48.71
38	Septum transparency	SeptTr	Code	1	5	3.29	1.24	37.72
39	Aril length	ArLe	mm	8.98	12.35	10.37	0.64	6.21
40	Aril width	ArWi	mm	4.81	7.57	6.53	0.61	9.28
41	Total aril weight per fruit	ToArWe	G	51.55	238.97	82.28	27.68	33.64
42	Aril shape	ArSh	Code	1	7	2.74	2.12	77.52
43	Aril color	ArCo	Code	1	9	6.63	2.02	30.51
44	Seed length	SeLe	mm	5.98	7.40	6.77	0.26	3.86
45	Seed width	SeWi	mm	1.85	3.18	2.56	0.22	8.71
46	Seed hardness	SeHa	Code	1	5	3.43	1.63	47.49
47	Fruit juice color	FrJuC	Code	1	7	4.37	1.94	44.44
48	Total soluble solids	TSS	%	14.00	23.00	19.06	1.65	8.66
49	Fruit taste	FrTa	Code	1	5	4.40	0.98	22.36
50	Fruit quality	FrQ	Code	1	7	4.91	1.72	34.97

**TABLE 2:** Frequency distribution for the measured qualitative morphological characters in the studied pomegranate genotypes

Character	Frequency (No. of genotypes)					
	0	1	3	5	7	9
Tree growth habit	‐	Spreading (12)	Semi‐erect (20)	Erect (38)	‐	‐
Tree growth vigor	‐	‐	Moderate (20)	High (50)	‐	‐
Shoot color	‐	Gray (35)	Gray brown (26)	Light brown (5)	Brown (4)	‐
Tree height	‐	Low (5)	Moderate (24)	High (41)	‐	‐
Trunk type	‐	Single‐trunk (17)	Multi‐trunk (52)	‐	‐	‐
Trunk diameter	‐	Low (8)	Moderate (60)	High (2)	‐	‐
Canopy density	‐	‐	Moderate (5)	High (65)	‐	‐
Tendency to produce suckers	‐	Low (46)	Moderate (11)	High (13)	‐	‐
Leaf upper surface color	‐	Light green (21)	Green (49)	‐	‐	‐
Leaf lower surface color	‐	Light green (53)	Green (17)	‐	‐	‐
Yield	‐	‐	Moderate (10)	High (60)	‐	‐
Fruit shape	‐	Round (58)	Oval (12)	‐	‐	‐
Fruit symmetry	Absent (15)	Present (55)	‐	‐	‐	‐
Stamen density in fruit calyx	‐	Low (16)	Moderate (22)	High (32)	‐	‐
Fruit calyx form	‐	Close (6)	Semi‐open (20)	Open (44)	‐	‐
Fruit peel color	‐	Yellow (10)	Yellow‐red (44)	Red (10)	Red‐brown (6)	‐
Fruit peel sunburn presence	Absent (49)	Low (8)	Moderate (13)	‐	‐	‐
Fruit peel cracking presence	Absent (67)	Present (3)	‐	‐	‐	‐
Fruit peel thickness	‐	Low (30)	Moderate (31)	High (9)	‐	‐
Internal peel color	‐	Cream (57)	Yellow (13)	‐	‐	‐
Septum color	‐	Glassy (18)	Milky (38)	Cream (14)	‐	‐
Septum thickness	‐	Low (24)	Moderate (40)	High (6)	‐	‐
Septum transparency	‐	Low (9)	Moderate (42)	High (19)	‐	‐
Aril shape	‐	Oval (35)	Stretched (18)	Triangular (8)	Prismatic (9)	‐
Aril color	‐	Light milky (2)	Pink (5)	White‐red (16)	Red (28)	Red‐black (19)
Seed hardness	‐	Soft (17)	Semi‐soft (21)	Hard (32)	‐	‐
Fruit juice color	‐	Colorless (6)	Pink (29)	Red (16)	Crimson (19)	‐
Fruit taste	‐	Sour (1)	Sour‐sweet (19)	Sweet (50)	‐	‐
Fruit quality	‐	Low (5)	Moderate (12)	High (34)	Very High (19)	‐

### 2.3 Statistical analysis

Analysis of variance (ANOVA) was performed to evaluate the variation among the genotypes based on the traits measured using SAS software (SAS Institute, 1990). Simple correlations between traits were determined using Pearson correlation coefficients (SPSS Inc., Norusis, 1998). Principal component analysis (PCA) was used to investigate the relationship between genotypes and to determine the main traits useful in the genotype segregation with SPSS software. Hierarchical cluster analysis (HCA) was performed using Ward's method and Euclidean coefficient with PAST software (Hammer et al., 2001). The first and second principal components (PC1/PC2) were used to create a scatter plot with PAST software.

## 3 RESULTS AND DISCUSSION

### 3.1 Morphological and pomological description

Most of the characters measured showed significant differences among the genotypes. These results were confirmed by CV so that 35 out of 50 characters measured exhibited the CVs more than 20.00%. The lowest CV belonged to seed length (3.86%) and then sepal number (4.19%), aril length (6.21%), fruit length (8.06%), fruit diameter (8.17%), TSS (8.66%), seed width (8.71%), and aril width (9.28%). In contrast, fruit peel cracking presence and fruit peel sunburn presence showed the highest CV (510.00% and 173.73%, respectively) (Table 1).

Firstly, 14 vegetative‐related characters were recorded to evaluate the variability among the genotypes. Tree growth habit was erect in most of the genotypes (38). Tree growth vigor, tree height, and canopy density were high in most of the genotypes (50, 41, and 65, respectively). Shoot color was gray in half of the genotypes (35). The tendency to produce suckers was low in most of the genotypes (46) (Table 2). Leaf length ranged from 43.64 to 79.81 mm, and leaf width varied from 11.95 to 20.79 mm. Petiole length varied from 3.15 to 7.61 mm, and petiole diameter ranged from 0.34 to 1.44 mm (Table 1).

Secondly, 36 fruit‐related characters were recorded to evaluate the variability among the genotypes. Yield was predominantly high (60). Fruit shape was round in 58, and fruit was symmetric in 55 genotypes. Fruit calyx length ranged from 11.72 to 30.22 mm, and fruit calyx diameter varied from 10.20 to 24.75 mm. Fruit calyx showed three forms, including close (6 genotypes), semi‐open (20), and open (44). The calyx form is a special and unique trait for each genotype. This information is beneficial for the design and selection of proper packaging for fruit storage and handling (Valero & Ruiz‐Altisent, 2000).

Fruit length varied from 70.37 to 113.30 mm, and fruit diameter ranged from 56.72 to 90.20 mm. Fruit weight ranged from 103.28 to 407.59 g, with an average of 158.04. Fruit size is essential in attracting consumer attention in the fresh food market. The presence of variation in fruit weight is mainly influenced by genetics, but pedo‐climatic conditions can also affect this trait (Martinez et al., 2006). In the present study, since all the genotypes were examined in the same geographical area, variation in fruit weight was more related to genetics.

Fruit peel weight ranged from 40.14 to 156.83 g, with an average of 68.54 (Table 1). Fruit peel color was highly variable and included yellow (10 genotypes), yellow‐red (44), red (10), and red‐brown (6) (Table 2). Peel color is one of the important criteria that farmers use this character to determine the appropriate harvest time (Melgarejo‐Sanchez et al., 2015). Besides, peel and aril colors are important traits related to fruit quality in pomegranate marketing (Mena et al., 2011). However, there is no significant positive correlation between peel color and aril color (Al‐Said et al., 2009). The attractive red color of the peel in pomegranate fruit is one of the main parameters for commercial quality grading that has a significant impact on consumer attention and choice (Zaouay & Mars, 2014).

The sunburn and cracking disorders were not observed on the peel of the majority of genotypes (49 and 67, respectively). Fruit peel thickness was low (30 genotypes), moderate (31), and high (9). The range of aril length and width was 8.98–12.35 mm and 4.81–7.57 mm, respectively. Aril size is most important for juice and fresh consumption of pomegranate fruit (Ferrara et al., 2011). Total aril weight per fruit ranged from 51.55 to 238.97 g, with an average of 82.28. Total yield of pomegranate aril is one of the most important criteria for industrial production of pomegranate (Maestre et al., 2000).

Aril shape showed high diversity, including oval (35 genotypes), stretched (18), triangular (8), and prismatic (9). Also, Aril color was highly variable, including light milky (2 genotypes), pink (5), white‐red (16), red (28), and red‐black (19). One of the most important criteria to attract the attention of consumers in pomegranate is the attractive red color of arils (Mena et al., 2011). Fruit juice showed four colors, including colorless (6 genotypes), pink (29), red (16), and crimson (19).

**FIGURE 1:**
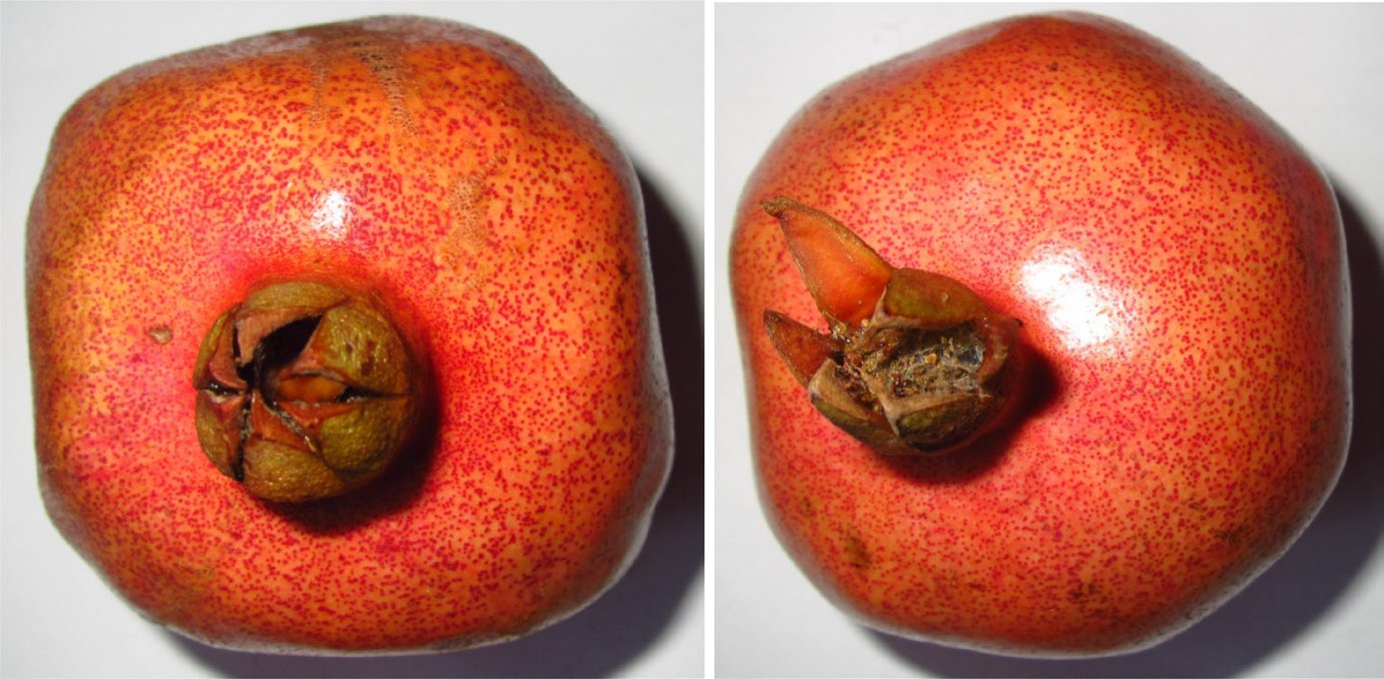
The fruit's pictures of two superior selected genotypes of pomegranate

Seed was soft in 17, semi‐soft in 21, and hard in 32 genotypes. Soft‐seeded pomegranates have a better flavor and taste than others and therefore are more popular in the market. In soft‐seeded pomegranates, testa width is thinner, seed and testa densities are lower, and the ratio of testa weight to total seed yield is lower (Prohit, 1985). The heritability of soft seededness in pomegranate is unknown, but it has been found that testa hardness increases in the hybrids obtaining from the crosses between soft‐seed genotypes and hard‐seed genotypes or soft‐seed genotypes (Prohit, 1987). Fruit taste was predominantly sweet (50 genotypes). The TSS ranged from 14.00 to 23.00%, with an average of 19.06. Therefore, since the TSS value was more than 12%, the juice extracted from the fruits in all genotypes is suitable for commercial uses (Vazquez‐Araujo et al., 2014). The fruit's pictures of two superior selected genotypes of pomegranate are shown in Figure 1.

### 3.2 Correlations among the measured characters

There were significant correlations between some characters (data not shown). Leaf length was positively and significantly correlated with leaf width (*r* = 0.57) and petiole length (*r* = 0.63). Fruit weight exhibited positive and significant correlations with fruit length (*r* = 0.79), fruit diameter (*r* = 0.94), and fruit stalk diameter (*r* = 0.60), and was in line with the previous findings in pomegranate (Karimi & Mirdehghan, 2013; Khadivi et al., 2018, 2020; Khadivi‐Khub et al., 2015). Fruit peel sunburn presence exhibited negative and significant correlations with tree growth habit (*r* = −0.30), tree height (*r* = −0.26), trunk diameter (*r* = −0.33), and yield (*r* = −0.39), while it was positively and significantly correlated with fruit peel color (*r* = 0.55) and fruit peel thickness (*r* = 0.36). Total aril weight per fruit was positively and significantly correlated with fruit length (*r* = 0.64), fruit diameter (*r* = 0.87), fruit weight (*r* = 0.95), fruit stalk diameter (*r* = 0.52), fruit peel weight (*r* = 0.71), and aril shape (*r* = 0.32). There was a positive and significant correlation between seed hardness and aril length (*r* = 0.25) and was in line with the previous findings in pomegranate (Karimi & Mirdehghan, 2013; Khadivi et al., 2018, 2020; Khadivi‐Khub et al., 2015). Fruit taste was positively and significantly correlated with aril length (*r* = 0.43), aril width (*r* = 0.43), seed width (*r* = 0.26), and TSS (*r* = 0.52). Fruit quality exhibited positive and significant correlations with aril color (*r* = 0.74), fruit juice color (*r* = 0.81), and TSS (*r* = 0.34) and was in line with the previous findings in pomegranate (Karimi & Mirdehghan, 2013; Khadivi et al., 2018, 2020; Khadivi‐Khub et al., 2015; Zamani et al., 2013). Estimating the correlation between morphological traits provides useful information for breeders that they can use in designing a high‐performance design to study genotypes (Tancred et al., 1995). This coefficient can also allow the comparison of direct and indirect selections and establish a strategy for the traits that are difficult to select and study (Falconer & Mackay, 1996).

### 3.3 PCA and HCA

The PCA exhibited 15 PCs, justifying 78.61% of the total variance (Table 3). Six characters, including fruit length, fruit diameter, fruit weight, fruit stalk diameter, fruit peel weight, and total aril weight per fruit, were correlated with PC1 and accounted for 11.77% of the total variance. The PC2 was associated with fruit calyx form, fruit peel color, fruit calyx length, fruit calyx diameter, aril length, aril width, TSS, and fruit taste, accounting for 9.96% of the total variance. The traits, including aril color, fruit juice color, and fruit quality, were correlated with PC3, explaining 7.81% of the total variance. It has been confirmed that fruit‐related traits were important for determining differences between genotypes and the selection of plant materials for use in pomegranate breeding programs (Karimi & Mirdehghan, 2013; Khadivi et al., 2018; Khadivi et al., 2020; Khadivi‐Khub et al., 2015; Zamani et al., 2013).

**TABLE 3:** Eigenvalues of the principal component axes from the PCA of morphological characters in the studied pomegranate genotypes

Character	Component														
	1	2	3	4	5	6	7	8	9	10	11	12	13	14	15
Tree growth habit	−0.18	0.07	−0.06	−0.03	0.27	0.00	−0.09	−0.74^a^	0.07	0.12	0.02	−0.03	−0.11	−0.07	0.05
Tree growth vigor	−0.26	−0.09	0.03	−0.25	0.29	−0.12	0.23	−0.10	0.20	0.20	0.42	−0.04	−0.15	−0.12	−0.16
Shoot color	0.04	−0.11	−0.14	−0.11	0.07	−0.18	−0.17	−0.06	−0.02	0.23	−0.64^a^	−0.02	−0.13	0.02	0.04
Tree height	−0.25	−0.03	0.07	−0.01	0.80^a^	0.04	−0.03	−0.14	0.07	0.26	0.13	−0.07	−0.10	0.04	−0.02
Trunk type	−0.23	0.18	0.07	0.14	−0.09	0.17	−0.05	0.38	−0.15	0.12	0.33	0.15	0.13	−0.03	−0.42
Trunk diameter	−0.36	0.18	−0.10	−0.07	0.58^a^	0.09	0.08	−0.40	0.02	−0.09	−0.04	−0.04	−0.04	0.11	0.12
Canopy density	−0.42	0.07	−0.02	0.00	0.73^a^	−0.10	−0.17	−0.25	−0.02	0.05	−0.17	0.01	−0.08	0.05	0.14
Tendency to produce suckers	0.25	−0.01	−0.19	−0.05	−0.70^a^	−0.12	−0.14	−0.17	0.02	0.28	0.07	0.17	−0.07	0.04	−0.03
Leaf length	0.09	0.13	0.12	−0.01	0.07	0.84^a^	−0.02	0.12	0.11	−0.11	0.11	0.03	0.10	−0.04	−0.06
Leaf width	0.05	−0.11	−0.04	0.05	−0.07	0.81^a^	0.21	−0.11	0.12	0.15	−0.02	−0.05	−0.01	0.07	−0.07
Leaf upper surface color	−0.10	−0.04	−0.07	−0.19	−0.21	−0.10	−0.06	0.09	0.06	0.01	0.35	−0.43	0.44	0.04	−0.18
Leaf lower surface color	−0.08	0.08	−0.09	0.10	−0.03	−0.07	−0.82^a^	−0.04	−0.02	0.01	0.00	−0.05	−0.09	0.18	0.04
Petiole length	−0.07	0.51	0.21	0.12	0.27	0.54^a^	0.00	0.11	0.05	−0.18	0.13	0.03	0.14	−0.14	0.02
Petiole diameter	−0.46	0.21	0.03	0.17	0.23	0.11	0.22	0.06	0.34	0.14	0.01	0.12	0.34	0.12	0.03
Yield	−0.16	0.31	0.23	−0.02	−0.05	0.17	0.66^a^	0.11	−0.01	−0.07	0.20	0.12	−0.07	0.14	0.06
Fruit length	0.86^a^	−0.13	−0.20	0.08	−0.06	−0.07	0.06	0.12	0.05	0.17	−0.10	−0.09	−0.02	0.07	−0.15
Fruit diameter	0.91^a^	−0.01	0.02	−0.05	−0.20	−0.03	−0.05	−0.05	0.08	0.03	−0.01	0.11	−0.04	0.05	−0.01
Fruit weight	0.94^a^	0.02	0.03	−0.09	−0.21	0.07	−0.04	0.03	0.03	0.05	0.05	0.09	0.03	0.01	−0.03
Fruit stalk length	0.19	−0.11	0.11	0.04	0.22	0.05	−0.11	−0.03	0.70^a^	0.10	−0.05	−0.11	−0.06	−0.30	0.09
Fruit stalk diameter	0.68^a^	−0.12	−0.05	−0.10	0.01	0.16	0.11	0.24	−0.05	−0.25	−0.09	−0.12	0.10	−0.03	0.05
Fruit shape	0.05	0.16	−0.01	−0.03	0.03	−0.03	−0.05	−0.03	0.03	0.84^a^	−0.19	−0.05	−0.06	0.01	−0.02
Fruit symmetry	0.10	0.04	0.09	−0.14	−0.15	−0.03	0.11	0.08	−0.02	−0.02	0.10	0.78^a^	0.04	0.02	0.07
Stamen density in fruit calyx	0.01	0.49	0.26	0.11	−0.21	0.14	0.29	0.15	−0.13	0.32	−0.11	0.17	0.23	−0.10	0.08
Fruit calyx form	−0.09	0.57^a^	−0.05	0.03	0.06	−0.10	0.38	−0.13	0.15	0.00	0.10	−0.23	−0.30	0.20	0.05
Fruit peel color	0.17	−0.54^a^	−0.37	−0.05	0.10	−0.25	−0.35	0.12	0.20	0.06	−0.09	0.05	−0.16	0.08	0.01
Fruit peel sunburn presence	0.00	−0.46	−0.40	0.17	−0.22	−0.18	−0.22	0.34	0.10	−0.07	−0.19	−0.08	0.15	−0.15	−0.03
Fruit peel cracking presence	0.07	−0.04	0.04	−0.17	−0.15	0.21	0.06	−0.11	0.72^a^	−0.13	0.16	0.04	0.06	−0.04	0.03
Fruit peel thickness	−0.06	−0.13	−0.32	0.45	−0.07	0.01	0.26	0.04	−0.12	0.17	−0.51	−0.26	0.12	−0.12	−0.14
Sepal number	−0.06	−0.25	−0.19	−0.05	0.21	−0.17	0.07	−0.14	−0.01	−0.02	0.14	−0.45	0.04	−0.52^a^	0.29
Sepal length	0.23	−0.51	−0.49	0.27	0.12	−0.14	0.25	−0.02	0.05	0.08	−0.18	0.01	−0.18	0.13	−0.12
Sepal base width	−0.19	0.36	0.03	−0.15	0.06	0.03	0.06	0.17	0.55^a^	0.16	−0.09	0.03	0.02	0.38	0.19
Fruit calyx length	0.22	−0.54^a^	−0.45	0.33	0.17	−0.21	0.07	0.13	0.07	0.14	−0.06	−0.16	−0.09	0.11	−0.15
Fruit calyx diameter	0.35	−0.71^a^	−0.38	0.21	0.06	−0.15	−0.06	−0.13	0.08	−0.03	−0.18	−0.10	−0.02	−0.01	−0.05
Fruit peel weight	0.93^a^	−0.12	−0.08	0.16	−0.08	−0.02	0.01	−0.01	−0.01	0.02	−0.15	0.06	0.08	0.00	−0.10
Internal peel color	0.12	−0.01	0.06	−0.07	−0.04	0.13	0.05	0.04	0.01	−0.09	0.03	0.04	0.78^a^	0.17	0.15
Septum color	−0.03	−0.01	−0.11	0.91^a^	0.02	0.03	−0.02	−0.05	−0.07	−0.02	0.03	−0.13	−0.11	0.05	−0.03
Septum thickness	−0.09	−0.08	−0.08	0.88^a^	−0.02	0.01	−0.09	0.05	−0.19	0.06	−0.02	0.04	0.08	0.06	0.07
Septum transparency	0.05	0.05	0.09	−0.94^a^	−0.02	−0.04	0.02	−0.03	−0.10	0.09	0.00	−0.04	0.06	−0.01	−0.01
Aril length	−0.06	0.64^a^	0.14	0.03	0.11	0.01	0.02	−0.10	−0.13	0.42	0.19	−0.01	0.16	0.22	0.31
Aril width	0.12	0.69^a^	0.10	0.03	−0.01	−0.08	0.10	−0.23	−0.12	0.21	0.11	0.22	0.15	0.12	−0.17
Total aril weight per fruit	0.83^a^	0.14	0.09	−0.24	−0.27	0.11	−0.07	0.06	0.06	0.06	0.16	0.11	0.01	0.00	0.03
Aril shape	0.25	−0.15	0.14	−0.06	−0.01	0.15	0.14	0.54^a^	0.07	0.41	0.30	0.08	−0.21	−0.12	0.17
Aril color	0.01	0.10	0.90^a^	−0.05	0.05	0.09	0.09	−0.07	0.07	0.06	−0.04	0.12	0.07	0.06	−0.09
Seed length	−0.25	0.02	−0.10	0.06	0.08	−0.10	−0.03	0.00	0.14	0.03	−0.02	0.11	0.15	0.08	0.83^a^
Seed width	−0.07	0.28	−0.11	0.33	0.45	−0.18	−0.05	−0.20	0.11	−0.07	0.04	0.43	0.22	−0.24	0.00
Seed hardness	0.09	0.04	0.11	0.13	0.10	−0.06	−0.11	−0.06	−0.15	−0.01	0.02	−0.05	0.23	0.72^a^	0.14
Fruit juice color	−0.04	0.12	0.90^a^	−0.02	0.09	0.06	0.11	0.10	0.09	−0.04	0.09	−0.01	−0.04	0.10	−0.04
Total soluble solids	0.09	0.61^a^	0.22	−0.11	0.28	−0.10	−0.10	0.34	0.04	−0.10	−0.16	0.07	−0.26	−0.14	−0.12
Fruit taste	0.00	0.78^a^	−0.11	−0.02	0.10	−0.05	−0.15	−0.06	0.21	0.05	−0.17	−0.02	−0.13	0.10	−0.09
Fruit quality	−0.03	0.10	0.85^a^	−0.18	0.00	−0.07	0.07	0.13	0.01	0.03	0.09	0.01	0.00	0.02	−0.02
Total	5.89	4.98	3.91	3.49	3.15	2.28	2.05	1.94	1.85	1.82	1.72	1.65	1.65	1.50	1.44
% of Variance	11.77	9.96	7.81	6.99	6.29	4.56	4.10	3.88	3.70	3.64	3.43	3.31	3.30	3.00	2.88
Cumulative %	11.77	21.73	29.55	36.53	42.82	47.38	51.48	55.35	59.05	62.69	66.12	69.43	72.73	75.73	78.61

^a^ Eigenvalues are significant ≥0.52.

**FIGURE 2:**
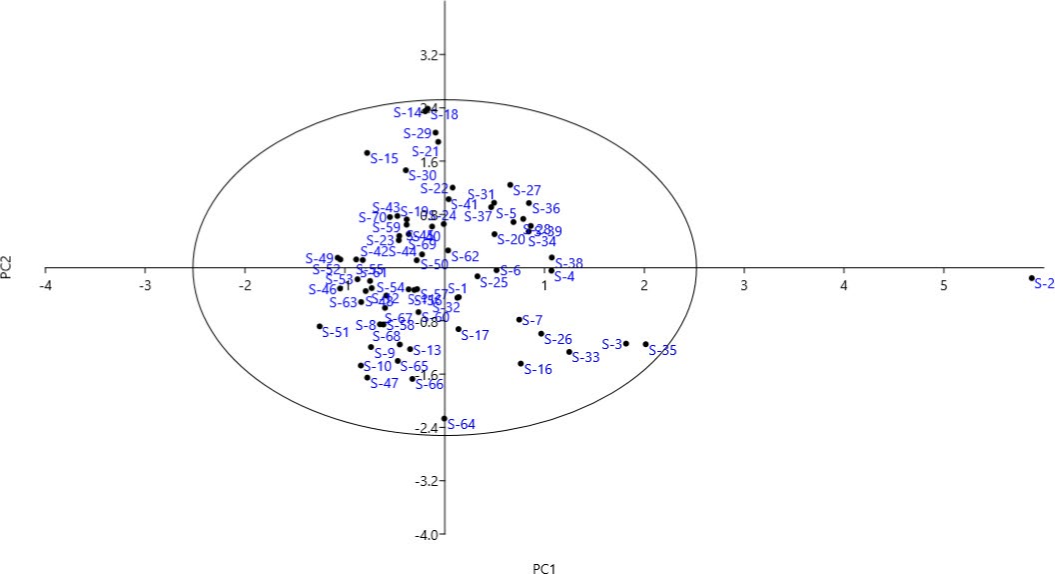
Scatter plot for the studied pomegranate genotypes based on PC1/PC2

The scatter plot of the genotypes was created based on effective traits in the PC1 and PC2 (Figure 2). The genotypes varied significantly in the PC1 in terms of fruit length, fruit diameter, fruit weight, fruit stalk diameter, fruit peel weight, and total aril weight per fruit. In the PC2, the genotypes showed a gradual increase in fruit calyx form, fruit peel color, fruit calyx length, fruit calyx diameter, aril length, aril width, TSS, and fruit taste.

**FIGURE 3:**
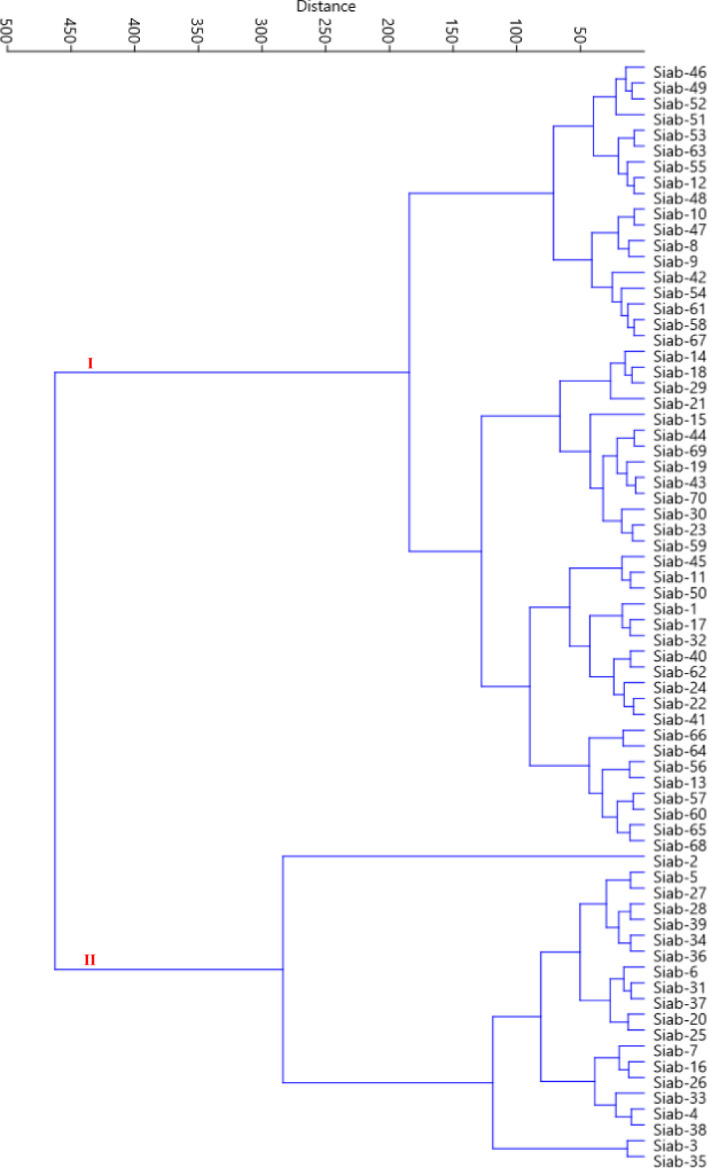
Ward cluster analysis of the studied pomegranate genotypes based on morphological traits using Euclidean distances

The HCA based on Ward's method divided the genotypes into two major clusters (Figure 3). The first cluster (I) contained 50 genotypes, which were placed into two sub‐clusters. Sub‐cluster I‐A included 18 genotypes, characterized by high values for fruit length, fruit diameter, fruit weight, fruit peel weight, and total aril weight per fruit. Sub‐cluster I‐B contained 32 genotypes, characterized by moderate values for the above characters. The rest 20 genotypes were classified into the second cluster (II), characterized by low values for the mentioned characters.

Considerable variation was detected between genotypes by PCA and HCA, the main reason being recombination (due to outcrossing) together seed propagation for long‐term as well as open‐pollination, and uncontrolled distribution of plant material (Jalikop & Sampath‐Kumar, 1990). The studied genotypes had a significant variability in different traits, many of which are related to genetics (Jalikop & Sampath‐Kumar, 1990), because the evaluations were performed in the same geographical area.

## 4 CONCLUSION

The goals of pomegranate breeding programs implemented in different parts of the world include achieving perfect vegetative growth, adequate flowering and fruiting, high and regular yield, large fruit size, red peel pool, red and soft aril, and excellent taste. Besides, no fruit cracking, an extended range of ripening, including early, medium and late, high and attractive juice, and sour, sweet, and sour‐sweet taste, are considered to choose the desired fruits pomegranates (Muradoglu et al., 2006). The current results showed significant variability among the genotypes. Based on the ideal values of commercial characters of pomegranate, including high yield, high fruit weight, red aril color, soft seed, and fruit taste, 15 genotypes, including Siab‐2, Siab‐35, Siab‐3, Siab‐33, Siab‐38, Siab‐4, Siab‐26, Siab‐39, Siab‐36, Siab‐34, Siab‐28, Siab‐16, Siab‐5, Siab‐7, and Siab‐27, were promising and could be directly cultivated in the orchards and used in the breeding programs.

## CONFLICT OF INTEREST

The authors declare no conflict of interest.

## RESEARCH INVOLVING HUMAN PARTICIPANTS AND/OR ANIMALS

None.

## INFORMED CONSENT

None.

## DATA AVAILABILITY STATEMENT

The data that support the findings of this study are available from the corresponding author upon reasonable request.
